# Impact of COVID-19 on cardiology services in a district hospital and adapting to the new normal

**DOI:** 10.1136/postgradmedj-2020-138228

**Published:** 2020-08-19

**Authors:** Saad Hasan, Haseeb Ur Rahman, Anish Patil, Carla Lewis, Ciaran Haye, Samuel Townsend, Stephen Hutchison

**Affiliations:** Cardiology, Aneurin Bevan University Health Board, Abergavenny, UK; Cardiology, Aneurin Bevan University Health Board, Abergavenny, UK; Cardiology, Aneurin Bevan University Health Board, Abergavenny, UK; Cardiology, Aneurin Bevan University Health Board, Abergavenny, UK; Cardiology, Nevill Hall Hospital, Abergavenny, UK; Cardiology, Aneurin Bevan University Health Board, Abergavenny, UK; Cardiology, Aneurin Bevan University Health Board, Abergavenny, UK

On March 23, 2020, in response to the COVID-19 outbreak, the UK government announced a series of measures (lockdown) to slow the spread of the virus and protect national health service (NHS) capacity. We describe the immediate effects on cardiology services in our hospital, comparing activity over the same period (March 23 to April 22) in 2019 and 2020.

Nevill Hall provides an acute cardiology service using 6 coronary care unit and 16 ward beds, non-invasive investigations and clinics. Routine angiography and pacing are currently undertaken at another hospital in the same health board on a shared waiting list, while acute coronary syndrome (ACS) patients are admitted here and have intervention at the local tertiary centre on a treat-and-repatriate basis.

There was a striking reduction in inpatient cardiology activity, particularly in the first week. Average patient numbers fell from 21 to 8. The other dramatic change was in heart failure presentations from 10 per month to 2. Throughout this period, there was plenty of bed capacity as elective surgery was cancelled and there was a fall in all non-COVID presentations, as reported in the UK.^1^ There is no reason to believe that patients have been admitted to other units.

The British Society for Echocardiography produced clear guidance for provision of echocardiography during the pandemic^2^ which we have followed, restricting inpatient scans to essential situations. Outpatient scans stopped along with face-to-face clinics on April 3. The number of inpatient echocardiograms performed and reported by sonographers during the specified period dropped from 144 to 38 and outpatient echocardiograms from 233 to 9.

There were similar reductions in other investigations in line with national and European guidance. Elective direct current cardioversion lists for atrial fibrillation, outpatient rhythm monitoring and myocardial perfusion studies were suspended. Pacing checks have reduced sixfold.

Inpatient angiogram/percutaneous coronary intervention (PCI) undertaken for ACS fell from 30 to 6, a drop of 80%. ST elevation myocardial infarction (STEMI) patients are repatriated after primary PCI, typically three to six per month but none in this period. Such patients are brought directly to a central cardiac catheter laboratory; several have presented over the study period and were discharged home rather than to their local hospital. However, there has been a significant drop in presentations from our Health Board population.

Outpatient activity has been switched predominantly to telephone consultations. Most clinics had been set up as ‘one stop’ with investigations on the same day. Similarly, cardiac rehabilitation sessions, nurse-led heart failure clinics and home visits have all been switched to telephone calls.

These figures starkly demonstrate the effect on a single hospital unit raising concerns that potentially serious conditions go untreated. Although presentations have gradually increased in the past 2–3 weeks, numbers are still well below expected. We are starting to see patients with heart failure as a consequence of untreated STEMI several weeks ago. Although we have managed cases of pericarditis and myocarditis, numbers are small in comparison to the ‘missing’ activity.

The substantial fall in non-COVID presentations has been documented in several healthcare systems. Comprehensive data from Spain showed a 40% reduction in primary STEMI intervention, along with a marked reduction in other interventional cardiology activity during this pandemic.^3^ Similar declines have been noted in recent data from various other countries. Our findings build on a worrying trend of reduced cardiac presentations worldwide. We postulate that patients are avoiding admission due to fear of developing infection or concern that the hospital is too overwhelmed to care for them. Anecdotal reports support this as do some of our own cases.

A 66-year-old man was admitted on April 22, he had been experiencing recurrent episodes of short-lived cardiac chest pain for several weeks. Electrocardiography (ECG) showed a fully evolved inferior infarction. Echocardiogram confirmed an extensive inferior and lower septal thin-walled dyskinetic segment with laminar thrombus, suggestive of completed STEMI. Ejection fraction was 30%. Coronary angiogram confirmed occluded right coronary artery and minor left system disease. On specific questioning he recalled an episode of prolonged severe pain 3 weeks before admission but was ‘too scared’ to come to the hospital, only seeking help because of recurrent episodes.

Similarly, an 87-year-old man presented with a 2-week history of dyspnoea. ECG showed that he was in complete heart block. Despite feeling short of breath for 2 weeks, he delayed his visit to the hospital for as long as he could.

During the 2003 severe acute respiratory syndrome (SARS) outbreak in Taiwan, fear of catching the virus in the hospital had a major impact on visits.^4^ Our findings show that a similar fear about COVID-19 is likely to have surfaced.

Overall, the NHS has coped remarkably well—the increased intensive therapy unit capacity and reorganisation of services have worked. We have not witnessed the widely broadcast images of overwhelmed hospitals seen in some countries. However, it is understandable that patients would be reluctant to come in; the message that ‘the NHS remains open for business’ must continue.

Finally, there are major long-term challenges for the service in terms of dealing with the backlog of clinics, investigations and procedures, as well as adapting working patterns and practices. The continuing need for social distancing and use of personal protective equipment will inevitably change the way clinics and investigations are undertaken, thus reducing the capacity per session. We will need to embrace different ways of working such as telephone and video consultations and improve vetting of referrals and investigation requests. Significant challenges with virtual clinics have been learnt from previous disease outbreaks, such as underdiagnosis and loss to follow-up.^5^ Careful evidence-based planning should be implemented to minimise disruption to patient care.

Against this background, the current referral to treatment times are unrealistic and will need to be reconsidered.
